# Profiling the best-performing community medicine distributors for mass drug administration: a comprehensive, data-driven analysis of treatment for schistosomiasis, lymphatic filariasis, and soil-transmitted helminths in Uganda

**DOI:** 10.1186/s12916-019-1303-z

**Published:** 2019-03-28

**Authors:** Goylette F. Chami, Narcis B. Kabatereine, Edridah M. Tukahebwa

**Affiliations:** 1 Department of Pathology, University of Cambridge, Tennis Ct. Rd., Cambridge, CB2 1QP UK; 2 Vector Control Division, Bilharzia and Worm Control Programme, Uganda Ministry of Health, Kampala, Uganda

**Keywords:** Mass drug administration, Coverage, Sub-Saharan Africa, Schistosomiasis, Lymphatic filariasis, Soil-transmitted helminths, Compliance, Praziquantel, Albendazole, Ivermectin

## Abstract

**Background:**

The most prevalent neglected tropical diseases are treated through blanket drug distribution that is reliant on lay community medicine distributors (CMDs). Yet, treatment rates achieved by CMDs vary widely and it is not known which CMDs treat the most people.

**Methods:**

In Mayuge District, Uganda, we tracked 6779 individuals (aged 1+ years) in 1238 households across 31 villages. Routine, community-based mass drug administration (MDA) was implemented for schistosomiasis, lymphatic filariasis, and soil-transmitted helminths. For each CMD, the percentage of eligible individuals treated (offered and ingested medicines) with at least one drug of praziquantel, albendazole, or ivermectin was examined. CMD attributes (more than 25) were measured, ranging from altruistic tendencies to socioeconomic characteristics to MDA-specific variables. The predictors of treatment rates achieved by CMDs were selected with least absolute shrinkage and selection operators and then analyzed in ordinary least squares regression with standard errors clustered by village. The influences of participant compliance and the ordering of drugs offered also were examined for the treatment rates achieved by CMDs.

**Results:**

Overall, only 44.89% (3043/6779) of eligible individuals were treated with at least one drug. Treatment rates varied amongst CMDs from 0% to 84.25%. Treatment rate increases were associated (*p* value< 0.05) with CMDs who displayed altruistic biases towards their friends (13.88%), had friends who helped with MDA (8.43%), were male (11.96%), worked as fishermen/fishmongers (14.93%), and used protected drinking water sources (13.43%). Only 0.24% (16/6779) of all eligible individuals were noncompliant by refusing to ingest all offered drugs. Distributing praziquantel first was strongly, positively correlated (*p* value < 0.0001) with treatment rates for albendazole and ivermectin.

**Conclusions:**

These findings profile CMDs who treat the most people during routine MDA. Criteria currently used to select CMDs—community-wide meetings, educational attainment, age, years as a CMD, etc.—were uninformative. Participant noncompliance and the provision of praziquantel before albendazole and ivermectin did not negatively impact treatment rates achieved by CMDs. Engaging CMD friend groups with MDA, selecting CMDs who practise good preventative health behaviours, and including CMDs with high-risk occupations for endemic infections may improve MDA treatment rates. Evidence-based guidelines are needed to improve the monitoring, selection, and replacement of CMDs during MDA.

**Electronic supplementary material:**

The online version of this article (10.1186/s12916-019-1303-z) contains supplementary material, which is available to authorized users.

## Background

Neglected tropical diseases (NTDs) comprise a set of 20 distinct ailments, which predominantly affect individuals living in rural poor areas of low-income countries [1]. The most prevalent NTDs are schistosomiasis, lymphatic filariasis, and soil-transmitted helminths (STHs) [2]. These chronic infections afflict an estimated more than one billion people worldwide and, if left untreated, can cause debilitating and irreversible morbidity [2, 3]. Schistosomiasis may contribute to portal hypertension and hepatosplenomegaly for *Schistosoma mansoni* and *S. japonicum* and bladder cancer for *Schistosoma haematobium* [4]. Lymphatic filariasis may result in severe hydrocoeles (men) and lymphoedema [5]. Amongst STHs, hookworm can cause anaemia, diarrhoea, and protein malnutrition [6].

The most widely implemented treatment strategy for schistosomiasis, lymphatic filariasis, and STHs relies on lay healthworkers/volunteers from NTD-endemic communities. Mass drug administration (MDA) is a diagnosis-free annual/biannual distribution of single-dose, oral preventive chemotherapies within NTD-endemic areas [7]. Concerning the quantity of dosage forms donated by pharmaceutical companies and the number of at-risk people targeted/treated, MDA is the largest infectious disease treatment programme worldwide [3, 8]. However, at least 1/3^rd^ of at-risk individuals requiring treatment do not receive preventive chemotherapies [3]. The most common MDA implementation uses community medicine distributors (CMDs). CMDs are unpaid volunteers apart from reimbursement for the training received from the local Ministry of Health. Members of endemic villages nominate individuals to serve as CMDs who are then responsible for treating all village residents eligible for MDA.

The provision of treatment using CMDs has been evaluated with respect to varying procedures for nominating CMDs [9, 10], understanding incentives/motivations of CMDs [10–12], measuring participant drug uptake rates [13, 14], and more recently examining the influence of CMD social networks [15–18]. These studies [9–14] have contributed to the understanding of how to retain CMDs throughout multiple years of MDA using kinship structures, the high opportunity costs incurred by CMDs when distributing medicine instead of working to earn income, and the challenges of participant willingness to ingest medicines provided by CMDs. The evaluation of CMDs thus far also has revealed biases towards socioeconomically marginalized individuals [15, 19] and how tightly-knit CMD friendships positively affect the reach and speed of MDA [17]. However, the aforementioned evaluations focus on cross-sectional or village-level data. There remains, to our knowledge, no quantitative study identifying the best-performing CMDs by observable, e.g. socioeconomic attributes. The main reason for this knowledge gap is that there is a need to deconstruct treatment rates by individual CMD. In this study, we examine treatment rates by CMD and comprehensively profile all CMDs responsible for treating an estimated more than 40,000 people for MDA. We address the following question. Who are the CMDs who treat the most people?

## Methods

### Study area and MDA tracking

Routine MDA was tracked from mid-July to mid-August in 2016 for 31 villages with an estimated 41,582 people eligible for treatment (Additional file 1: supplementary methods). The villages were located within 5 km of Lake Victoria and across six sub-counties (at the time of survey) within Mayuge District. The same vector control officers and one district health officer oversaw the study villages and were responsible for the training and monitoring of CMDs. The sub-counties were chosen based on (1) current eligibility for the routine round of MDA studied here, (2) ongoing distribution of the same set of drugs during MDA, (3) ongoing implementation of community-based MDA, and (4) having had the same number of previous rounds of MDA [17]. The specific study villages were chosen due to the endemicity with intestinal schistosomiasis (*S. mansoni*) and hookworm; infection prevalence for individuals aged 5+ years was 36% and 41%, respectively [20]. Prevalence of lymphatic filariasis in the study area is less than 5% [21].

In 2003, Uganda was the first country to receive MDA—both school and community-based—for schistosomiasis [22]. Community-based MDA was provided to treat individuals with praziquantel for schistosomiasis and with albendazole and ivermectin for lymphatic filariasis. Albendazole was not donated for community-wide treatment of hookworm; however, individuals in our study area benefited from ongoing albendazole distribution for lymphatic filariasis [20]. At the time of this study, our villages had received at least 10 annual rounds of community-based MDA for schistosomiasis and STHs.

Two CMDs per village were tasked with treating all eligible individuals within their village, i.e. children (including both enrolled and non-enrolled schoolchildren) and adults. During the study period, school-based MDA had not yet begun (for schistosomiasis and STHs) and drugs were only available from CMDs. To emphasize, CMDs were responsible for administering all treatments for schistosomiasis, STHs, and lymphatic filariasis within their villages. The eligible treatment groups included school-aged children and adults with praziquantel for schistosomiasis; preschool-aged children, school-aged children, and adults with albendazole for STHs; and school-aged children and adults with albendazole and ivermectin for lymphatic filariasis.

The CMDs were trained to move from door-to-door to administer and observe ingestion of the required medicines. This procedure is the only nationally approved method of distribution for community-based MDA in the study area. Hence, community-based MDA here differs from community-directed MDA in that the village members are neither choosing how MDA is implemented, e.g. door-to-door versus central point distribution or a combination thereof, nor deciding the days/timing when MDA occurs. Community-based and community-directed MDA are similar with regard to one key aspect of community-wide MDA—both select CMDs through community-wide meetings. The CMDs were instructed by national programmes to administer praziquantel in week 1 and albendazole with ivermectin in week 2 of MDA. All CMDs were provided a sufficient number of all pills/tablets to treat everyone for the aforementioned infections in their village. To remove administrative barriers during MDA, vector control officers were provided with a car to ensure CMDs received timely training and initiated MDA at the same time [17]. Although the national programme instructs CMDs to administer treatment within 2 weeks, drugs are not retrieved from the villages after 2 weeks and drug delivery by CMDs predominantly occurs over at least a 1-month period in the study area [17]. Thus, to enable a comprehensive tracking of MDA, routine MDA proceeded undisturbed for 1 month as opposed to 2 weeks. After the 1-month period, surprise visits from survey teams were conducted in all villages [17, 19].

### Participant sampling

**Fig. 1:**
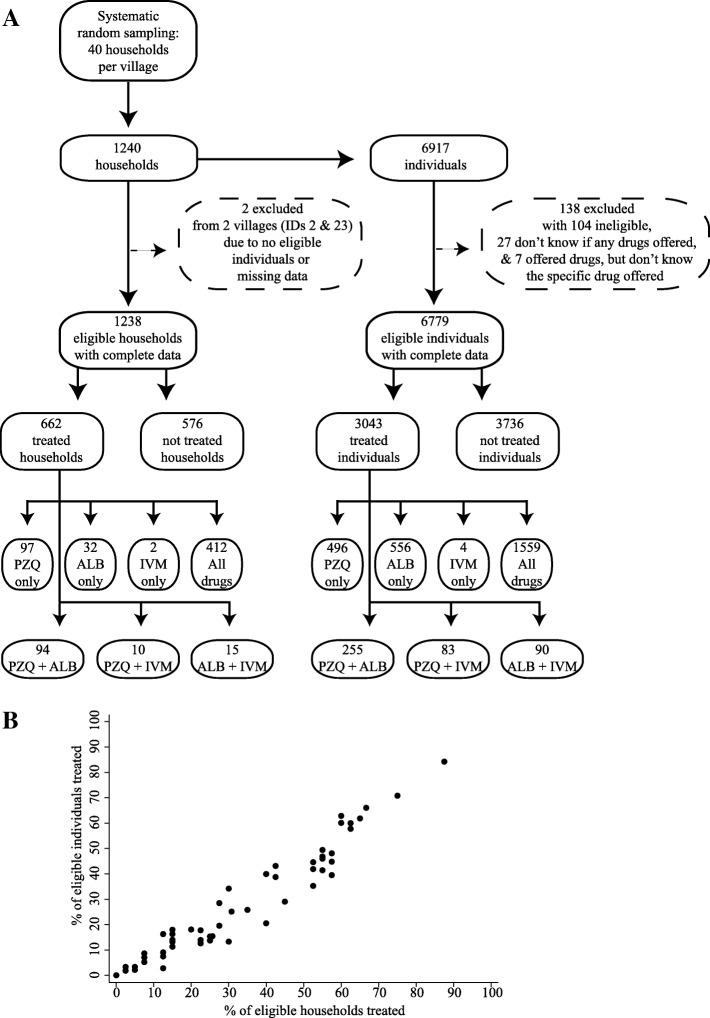
Treatment outcomes. PZQ praziquantel, ALB albendazole, IVM ivermectin. A treated individual was someone eligible for MDA and who was offered and ingested at least one drug of PZQ, ALB, or IVM from a CMD during 1 month of MDA. A treated household comprised at least one eligible person in the home who was offered and had ingested at least one drug of PZQ, ALB, or IVM from a CMD during 1 month of MDA. **a** Breakdown of treatment outcomes for 31 villages by household and individual. **b** Scatter plot of treatment outcomes for CMDs. *Pearson r =* 0.968, *p* < 0.0001

Forty households were systematically randomly sampled in each village using local registrers where all current residents were listed by household (Additional file 1: supplementary methods). As this study was data-driven, sample size calculations were conducted to achieve high precision and accuracy. We assumed there were 8000 households across the 31 study villages (final count was 7452 households) and treatment rates of 50% (conservative at the household level for our study area [17]). Accordingly, 613 households were needed to achieve a 99% confidence level with a 5% confidence interval. However, households were grouped by village, so to allow for a generous design effect, 613 was multiplied by two resulting in 1226 households required. A common denominator of 40 households per village was chosen to enable comparisons amongst CMDs across villages. Ultimately, 6779 individuals (all members of sampled households aged 1+ years) in 1238 households across 31 villages had complete data and were examined in this study (Fig. 1). Information on all CMDs—two per village—also was gathered. This analysis focused on the 59 CMDs (total possible = 62) with complete data. Additional details of MDA tracking and participant sampling are provided in Additional file 1: supplementary methods.

### Variables

Treatment rates for each CMD were calculated as the percentage of eligible individuals in the village—1+ years for albendazole and 5+ years for praziquantel/ivermectin—who were treated by the CMD of interest. Treatment comprised the offer of at least one drug of praziquantel, albendazole, or ivermectin by a CMD and the recipient’s ingestion of at least one offered drug (Fig. 1). For each treated individual, household survey respondents reported the main type of location of where drugs were received from CMDs. This categorical variable included the participants’ home, local health centres, primary schools, participants’ friends’ houses, CMDs’ houses, and the chairman’s (village leader’s) home. Participant noncompliance was measured for all drugs; it was defined as the offer of a drug to an eligible individual who refused to ingest the offered drug. For all eligible individuals who did not comply with at least one treatment or were not offered any treatment, the reasons from the perspective of the participant for noncompliance or no offer were recorded. No limit was placed on the number of reasons/responses from an individual. An eligible individual could have not complied with a treatment and still be classified as treated as long as the individual consumed at least one drug. Notably, the World Health Organization (WHO) treatment targets for the diseases of interest here are as follows: at least 75% treatment of at-risk individuals with praziquantel or albendazole, respectively, for schistosomiasis and STHs; and at least 65% of eligible individuals with both albendazole and ivermectin for lymphatic filariasis [7]. The number of CMDs who met these targets was described, although WHO targets are defined for communities and nonexistent for individual CMDs.

We examined who treated whom as follows. For each individual offered at least one drug, the name of the CMD who offered the drug(s), was recorded from the household survey respondent (Additional file 1: supplementary methods). If both CMDs offered a drug to an individual, then both CMDs’ names were recorded. Hence, the percentage of individuals treated by each CMD cannot necessarily be added together and equated to the percentage of individuals treated in the village. CMDs are selected by their fellow community members and thus are well known throughout their village [17]. No respondents indicated that they did not know the name of the two CMDs within their village. Similarly, no respondents reported that they did not know the name of the CMD(s) who offered drugs to a particular household member. To minimize any recall bias, respondents only provided information about those treated within their household and did not provide information on which drug each CMD provided to each individual. Thus, if an individual within a home was offered more than one drug by more than one CMD, then both CMDs were recorded as giving all drugs offered.

To assess intrinsic motivations, altruistic tendencies of CMDs were measured as follows. CMDs were asked to make a choice amongst three options of pre-divided sums of money. The choices provided to CMDs are shown in Tables 1 and 2. Each option had an amount of money that the CMD would receive and an amount of money another individual would receive. The highest payoff was 5500 Ugandan shillings, which was approximately equal to half of what CMDs would receive for remuneration for attending a full day of MDA training or the value of 1 day of casual labour in the study area [11]. The lowest payoff was 500 Ugandan shillings for the other individual. The amounts were chosen so that CMDs would make decisions about amounts that were not trivial, i.e. too low within the local context, or conversely were not too high to avoid decisions that would result in a major change in living standards of the CMD. Each option represented a different well-established social value orientation [23]. This task was repeated six times with each CMD. The number of altruistic choices was counted and, as described in van Lange [23], if a CMD chose an altruistic response at least five times, then the CMD was classified as altruistic. This game was conducted twice for each CMD where the identity of the other/receiving individual was either a complete stranger or a close friend (Tables 1 and 2). The complete protocol for the prosocial game and definition of social value orientations are provided in the Additional file 1: supplementary methods. Two variables were constructed from these games: baseline altruism and in-group bias. Baseline altruism was a binary variable of the CMD’s altruism classification towards strangers, and in-group bias was a binary variable equal to one if the CMD was classified as altruistic towards close friends, but not towards strangers.

**Table 1:** Prosocial games: stranger outside the village who you will never meet

	A	B	C
You	3500	4200	4000
Stranger	500	2000	3000
You	4000	5000	5300
Stranger	4000	1000	2000
You	5500	5000	5000
Stranger	3000	4500	1000
You	5400	4800	5000
Stranger	3000	1000	5000
You	4800	5400	4800
Stranger	4800	2400	800
You	5000	4500	5200
Stranger	500	4000	2200

**Table 2:** Prosocial games: one of your close friends

	A	B	C
You	4800	5400	4800
Close friend	4800	2400	800
You	5500	5000	5000
Close friend	3000	4500	1000
You	3500	4200	4000
Close friend	500	2000	3000
You	5000	4500	5200
Close friend	500	4000	2200
You	4000	5000	5300
Close friend	4000	1000	2000
You	5000	4800	5400
Close friend	5000	1000	3000

A wide range of CMD attributes was measured. The status-seeking behaviour of a CMD was measured as a binary variable equal to one if the CMD purposely sought at least one of their current close friendships with a person of formal (high) status [15]. Formal status was defined as currently holding or having previously held a position within the local council (village government), on the beach management committee, or as a religious/tribe/clan leader. CMDs were asked who nominated them to be a CMD, if/how their friends helped with MDA, and the number of years they had been active as a CMD. Eleven socioeconomic characteristics of CMDs also were calculated. To account for the placement of each CMD in their village social network, which has been shown to affect village-level treatment rates [17], the connections between CMDs and only their immediate friends were graphed. CMDs named up to 10 close friends and these friends were interviewed to elicit their relations with the CMD and other CMD-nominated individuals. Six network indicators were examined to assess the cohesiveness/sparseness of friendship connections. Comprehensive descriptions for all variables are provided in Additional file 1: supplementary methods, Table S1, Table S2, and Table S3.

### Statistical analysis

Data were analyzed in *R* v3.2.3, Stata v13.1, and Python v2.7.11. With a comprehensive set of 25 CMD characteristics and only 59 CMDs, the final set of variables used to predict treatment rates were chosen using a data-driven approach—the least absolute shrinkage and selection operator (Lasso)—described in [24]. For Lasso, ordinary least squares regression (OLS) was used with a variable penalization based on the absolute value of coefficients; variables with non-zero coefficients were kept and used as predictors of treatment rates achieved by CMDs [24]. Mixed models allowing for village-level random effects were compared to simple OLS models with a single intercept; there was no support for use of a mixed model (likelihood ratio test *p* value > 0.20). Thus, for the final model, OLS regressions were run with robust standard errors clustered by village to account for any village-level variation such as differences in the number of households per village or the compactness of the spatial dispersion of homes. To assess internal model validity, eight-fold cross-validation was run for both the variable selection and final OLS model.

## Results

### Treatment variation by CMD

Only 44.89% (3043/6779) of all individuals eligible for at least one drug were treated within 1 month of MDA (Obs. 62 CMDs). Only 11.73% (357/3043) of all treated individuals were offered drugs by both CMDs. In 51.61% (16/31) of villages, no eligible individuals were treated by both CMDs (Obs. 62 CMDs). Praziquantel was the most often administered medicine. Treatment rates for praziquantel, albendazole, and ivermectin were 40.99% (2393/5838), 36.29% (2460/6779), and 29.74% (1736/5838), respectively. The average treatment rate per CMD was low. Considering the conservative administration of at least one drug, CMDs treated an average of only 26.08% of eligible individuals (Obs. 59 CMDs, std. dev. 21.79%). These treatment rates varied widely across CMDs from 0% to 84.25% (Table 3); 10.17% (6/59) of CMDs did not treat a single person in their village. CMDs who treated many people did not simply approach a few large households. The percentage of individuals treated by CMDs was strongly positively correlated (*Pearson's* *r* = 0.968, *p* < 0.0001) with the percentage of households approached by CMDs (Fig. 1 and Additional file 1: supplementary methods).

**Table 3:** Treatment rates by drug and across community medicine distributors

Variable	Obs.	Mean (%)	Std. Dev. (%)	Min (%)	Max (%)
% eligible individuals treated with at least one of PZQ, ALB, or IVM	59	26.08	21.79	0	84.25%
% eligible individuals treated with PZQ	59	23.88	20.24	0	72.24
% eligible individuals treated with ALB	59	20.44	19.89	0	82.78
% eligible individuals treated with IVM	59	16.95	18.49	0	82.45

*PZQ* praziquantel, *ALB* albendazole, *IVM* ivermectin

### Participant noncompliance

**Fig. 2:**
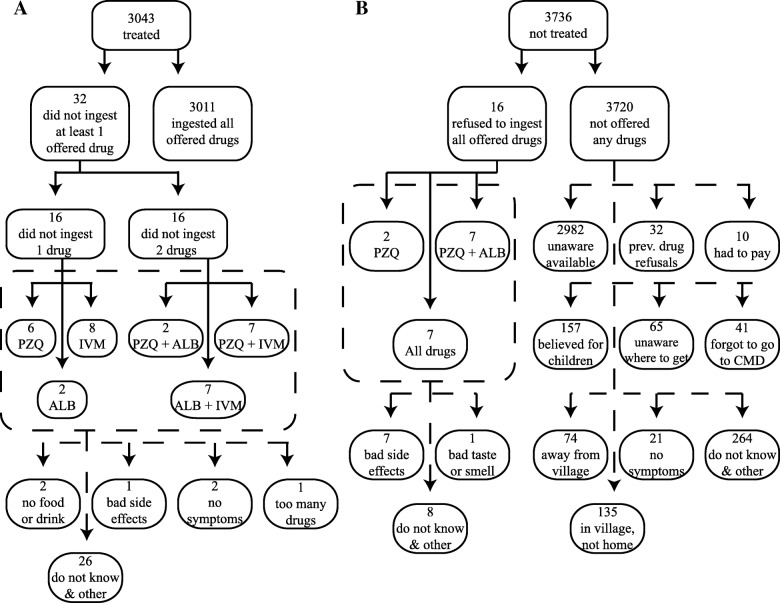
Reasons for refusal of treatment. PZQ praziquantel, ALB albendazole, IVM ivermectin. A treated individual was someone eligible for MDA and who was offered and ingested at least one pill of PZQ, ALB, or IVM from a CMD during 1 month of MDA. **a** A breakdown of the specific drugs and number of drugs that were offered, but not ingested amongst treated individuals is shown. Individuals did not provide reasons for refusing specific drugs. Hence, if a treated individual did not ingest more than one offered drug, then the reason of refusal applied to all of the drugs that were not ingested. Here, no more than one reason for refusing a drug was provided per individual, though no limit was imposed. **b** The number of individuals, amongst individuals eligible for MDA, and their reasons for either refusing to ingest all offered drugs or for not being offered any drugs are shown. Individuals who were offered drugs, but refused to ingest all of the drugs offered provided only one reason for refusing treatment. The eligible individuals who were not offered any drugs provided up to two reasons for not being offered treatment. No limits on the number of responses allowed were imposed

Noncompliance was infrequent and did not contribute to the low treatment rates achieved by CMDs. Figure 2 presents the breakdown of noncompliance by drug and reason. Only 0.24% (16/6779) of eligible individuals were noncompliant by refusing to ingest all offered drugs. Amongst treated individuals, a further 32 eligible individuals refused to ingest at least one of the offered medicines. Together with the systematic noncompliers, the frequency of noncompliance within an eligible population of 6779 individuals was only 0.71% (48/6779). Amongst these few noncompliers, only 31 individuals refused to ingest praziquantel and only nine individuals noted reasons of bad side effects or bad taste/smell of drugs. Although noncompliance was infrequent, the lack of information dissemination by CMDs was apparent (Fig. 2). Amongst the eligible individuals who were not approached and offered any drugs by CMDs, 80.16% (2982/3720) were unaware that any drugs were available within the treatment period.

**Fig. 3:**
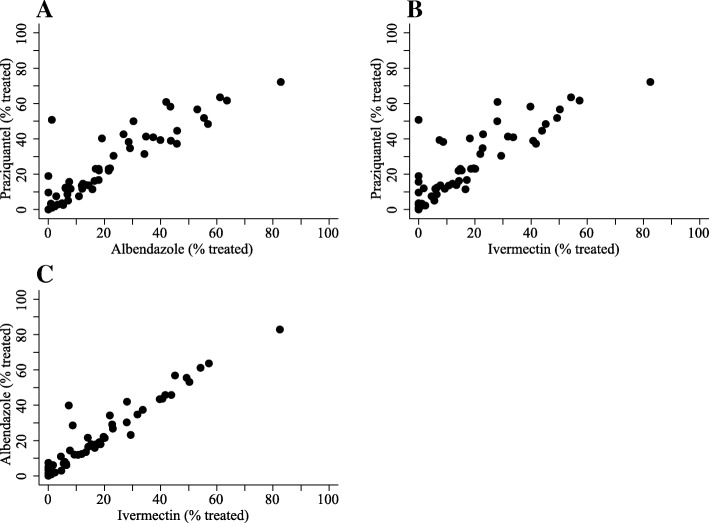
Correlation between drug-specific treatment rates. Treatment rates achieved by CMDs (59 obs.) are shown. Treatment rates were measured as the percentage of eligible individuals who were offered and had ingested the drug of interest. The drug-specific treatment rates, i.e. the different outcomes for a CMD, were strongly, positively correlated. **a** *Pearson’s r* = 0.898, *p* value < 0.0001. **b** *Pearson’s r* = 0.858, *p* value< 0.0001. **c** *Pearson’s r* = 0.959, *p* value < 0.0001

Any ordering effects of administering praziquantel first on the subsequent compliance with or offer of albendazole and ivermectin were investigated. Low rates of noncompliance with albendazole and ivermectin were observed for individuals who consumed praziquantel. Amongst the eligible individuals who consumed praziquantel and were offered albendazole, 0.49% (9/1823) did not ingest albendazole. Amongst the eligible individuals who consumed praziquantel and were offered ivermectin, 0.85% (14/1656) did not ingest ivermectin. Concerning drug-specific treatment rates, the distribution of praziquantel had a positive spillover effect and was strongly associated (*p* value < 0.0001) with the increased distribution of albendazole and ivermectin (Fig. 3).

### Method of drug distribution

When all treated individuals were asked where they were offered drugs, 85.44% (2600/3043) indicated at their home, i.e. from the nationally approved door-to-door method of drug distribution. There were 9.40% (286/3043) of treated individuals who retrieved drugs directly from a CMD’s home. Door-to-door drug distribution also was predominantly used when both CMDs treated the same individual; 81.79% (292/357) of these treated individuals were offered drugs at their home. Only 6.16% (22/357) of individuals treated by both CMDs had retrieved drugs from the CMDs’ homes. All treated individuals who did not receive drugs at their home or at a CMD’s home were offered treatment by CMDs at a central point, e.g. a local health centre (0.56%, 17/3043), primary school (1.81%, 55/3043), the chairman’s home (0.30%, 9/3043), or at a friend’s house (0.76%, 23/3043). Only 1.74% (53/3043) of treated individuals did not know the location of drug receipt.

### Who was a CMD?

Characteristics of CMDs are presented in Table 4. NTD-endemic communities selected CMDs, on average, who were old compared to the median age in the study villages (42.86 years; 88^th^ percentile of age) and were moderately educated, i.e. completed only 1 year of education after primary school. CMD turnover was low; individuals had been CMDs for an average of 8.71 years. Approximately 50% of CMDs were female or belonged to the majority tribe/religion of their village. Farming was the most common occupation (74.58%). A small minority of fishermen/fishmongers (8.47%) also were CMDs. The vast majority of CMDs engaged in good preventative health behaviours; 79.66% and 86.44% of CMDs, respectively, retrieved drinking water from a protected source and owned a private home latrine. CMDs were well established within their village; they belonged to a household that had resided in the village for many years (avg. 22.98) and 27.12% of CMDs held or had held formal positions of power on the local council (village government), as tribe/clan/religious leaders, or on the beach management committees. Although national MDA programmes instruct villages to nominate CMDs through open community meetings, only 44.07% of CMDs were selected in this manner. The remaining CMDs were selected directly by a local council member (50.85%) or by a village health team member (5.08%), which is the lowest administrative medical unit in Uganda.

**Table 4:** Characteristics of community medicine distributors

Variable	All CMDs	CMDs in ≤ 50^th^ percentile^b^	CMDs in > 50^th^ percentile^b^	CMDs who treated no one
	(*n* = 59)	(*n* = 30)	(*n* = 29)	(*n* = 6)
Baseline altruism, no. (%)	8 (13.56)	3 (10.00)	5 (17.24)	0 (0)
In-group bias, no. (%)	28 (47.46)	11 (36.67)	17 (58.62)	1 (16.67)
Sought friends w/formal (high) status, no. (%)	18 (30.51)	12 (40.00)	6 (20.69)	2 (33.33)
CMD selected in community meeting, no. (%)	26 (44.07)	11 (36.67)	15 (51.72)	3 (50.00)
CMD selected by local council (vill. gov.), no. (%)	30 (50.85)	17 (56.67)	13 (44.83)	2 (33.33)
CMD selected by village health team, no. (%)	3 (5.08)	2 (6.67)	1 (3.45)	1 (16.67)
CMD’s friends help with MDA, no. (%)	44 (74.58)	20 (66.67)	24 (82.76)	5 (83.33)
Years as CMD, mean (Std. Dev.)	8.71 (4.53)	8.07 (4.62)	9.38 (4.43)	5.5 (4.76)
Age, mean (Std. Dev.)	42.86 (10.07)	40.20 (8.08)	45.62 (11.26)	41 (9.21)
Female, no. (%)	30 (50.85)	18 (60.00)	12 (41.38)	4 (66.67)
Education, mean (Std. Dev.)	8.29 (1.91)	8.07 (1.82)	8.52 (2.01)	8.17 (1.60)
Majority tribe, no. (%)	30 (50.85)	15 (50.00)	15 (51.72)	4 (66.67)
Majority religion, no. (%)	31 (52.54)	17 (56.67)	14 (48.28)	4 (66.67)
Farmer, no. (%)^a^	44 (74.58)	25 (83.33)	19 (65.52)	4 (66.67)
Fisherman/fishmonger, no. (%)^a^	5 (8.47)	1 (3.33)	4 (13.79)	0 (0)
Formal status: current/former local council (village gov.) member, tribe/clan/religious leader, or beach management committee member, no. (%)	16 (27.12)	6 (20.00)	10 (34.48)	2 (33.33)
Household uses protected drinking water source, no. (%)	47 (79.66)	21 (70.00)	26 (89.66)	5 (83.33)
Household owns private home latrine, no. (%)	51 (86.44)	26 (86.67)	25 (86.21)	5 (83.33)
Home quality score, mean (Std. Dev.)^c^	9.66 (2.73)	9.60 (2.54)	9.72 (2.96)	9.5 (2.26)
Years since household settled in village, mean (Std. Dev.)	22.98 (11.29)	22.27 (10.88)	23.72 (11.85)	25.33 (10.88)

*CMD* community medicine distributor

^a^Base category is “other” occupations

^b^50^th^ percentile of treatment rates achieved by CMDs = 17.95%. Treatment rates = % of eligible individuals within CMD’s village who the CMD treated with at least one drug of praziquantel, albendazole, or ivermectin

^c^Sum of all rankings (1–4) of materials for roof, floor, and wall (Additional file 1: supplementary methods)

A summary of CMD network indicators is provided in Additional file 1: Table S3

Regarding the prosocial tendencies of CMDs, only 13.56% of CMDs were classified as altruistic. Nearly half of CMDs (47.46%) displayed in-group biases, indicating that CMDs are more likely to act altruistically towards their close friends than towards strangers. Almost one-third of CMDs had purposely sought at least one of their current close friendships to establish links to someone in their village with high (formal) status. There was little variation amongst CMDs with respect to the structure of their close friendships; most CMDs belonged to a network of dense, closely knit friends (Additional file 1: Table S3).

### Profiles of best-performing CMDs

**Fig. 4:**
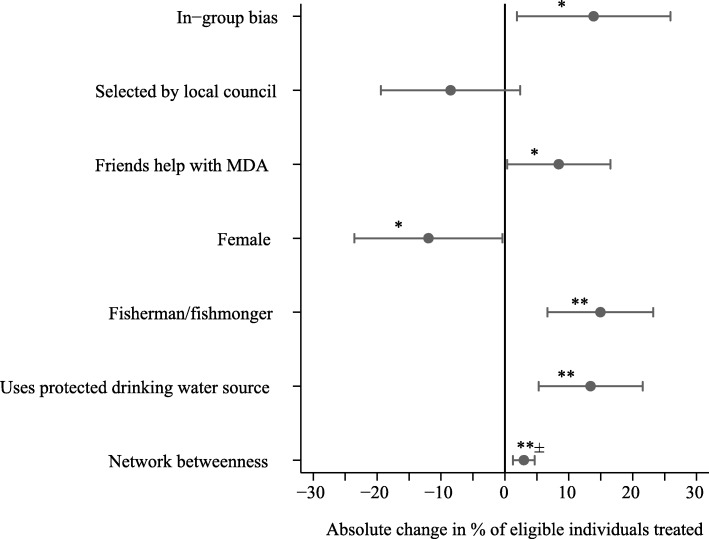
Determinants of treatment rates of community medicine distributors. This figure shows the increase/decrease in the percentage of eligible individuals treated with at least one drug of praziquantel, albendazole, or ivermectin by a CMD. The results are the coefficients of the linear regression shown in Table 5. *p value < 0.05, **p value < 0.01, ± Network betweenness was not significant (*p* value > 0.05) when one outlier was removed (Additional file 1: Table S4); 58/59 CMDs had network betweenness ≤ 3 whilst 1/59 CMDs had network betweenness = 11.58. In-group bias is positive if the CMD was altruistic towards their friends and not altruistic towards strangers

Only 28% (7/25) of the initial characteristics of CMDs were relevant for predicting CMD treatment rates, i.e. identified through the data-driven approach for variable selection and included in the linear regression (Fig. 4, Table 5, and Additional file 1: Table S4). The final significant (*p* value < 0.05) determinants of treatment concerned the friends, gender, occupation, and preventative health behaviours of CMDs. How a CMD viewed their friends and whether a CMD involved their friends with MDA was related to how many people the CMD treated. CMDs, who were more altruistic towards their friends than towards strangers (positive in-group biases), were predicted to treat 13.88% more individuals when compared to CMDs without in-group biases. If a CMD had friends who helped with MDA, then the CMD’s treatment rate was estimated to increase by 8.43% relative to the treatment rate of a CMD whose friends were not involved with MDA.

**Table 5:** Determinants of treatment rates by community medicine distributors

Variable^a^	Coef.	Clustered robust SE	*p* value	95% confidence interval	
In-group bias^b^	0.139	0.059	0.025	0.019	0.259
Selected by local council^c^	− 0.085	0.053	0.121	− 0.194	0.024
Friends help with MDA^d^	0.084	0.040	0.041	0.004	0.165
Female	− 0.120	0.057	0.043	− 0.235	− 0.004
Fisherman/fishmonger^e^	0.149	0.040	0.001	0.067	0.232
Household uses protected drinking water source	0.134	0.040	0.002	0.053	0.216
Network betweenness^f^	0.030	0.008	0.001	0.013	0.047
Constant	0.095	0.050	0.067	− 0.007	0.197

Obs. 59, *R*^2^ = 0.331, *F-stat.* 13.69, *F-stat. p* value < 0.0001. Root mean squared error (RMSE) from 8-fold cross-validation = 0.183. Variables selected through Lasso with 8-fold cross-validation. Mean squared error (MSE) of Lasso cross-validation = 0.076

^a^The results shown are from an ordinary least squares regression with standard errors clustered by the village

^b^In-group bias is positive if the CMD was altruistic towards their friends and not altruistic towards strangers

^c^The base category includes CMD selection by community meeting or direct nomination from a village health team member

^d^MDA = mass drug administration

^e^The base category for these occupations includes all other CMD occupations

^f^Network betweenness was not significant (*p* value > 0.05) when one outlier was removed (Additional file 1: Table S4) 58/59 CMDs had network betweenness ≤ 3 whilst 1/59 CMDs had network betweenness = 11.58

Three personal characteristics of CMDs were associated with treatment. Female CMDs were predicted to treat 11.96% fewer individuals than male CMDs. The discrepancy in treatment rates between female and male CMDs was not because CMDs only treated individuals of the same gender. Amongst the eligible individuals treated by female CMDs and male CMDs, 49.76% (611/1228) were male and 50.00% (967/1934) were female, respectively. Being a fisherman/fishmonger was associated with a 14.93% increase in the percentage of individuals treated when compared to the treatment rates of CMDs from any other occupation. If a CMD belonged to a household that retrieved drinking water from a protected source then the CMD was estimated to treat 13.43% more people when compared to a CMD whose household used unsafe water sources. All analyses were rerun at the household level where the outcome of interest was at least one eligible person in the home who was treated with at least one drug of praziquantel, albendazole, and ivermectin; all results remained robust (Additional file 1: Table S5 and Table S6). Concerning the CMDs who did not treat anyone, only two characteristics were shared. All six of the CMDs who treated no one were not fishermen/fishmongers and were not classified as altruistic individuals. CMD characteristics were associated with how many people CMDs chose to treat as opposed to which drugs CMDs chose to administer (Additional file 1: Table S7 and Table S8).

Remarkably, no CMD met the WHO treatment target for schistosomiasis, whereas only a single CMD (the same CMD) met the WHO treatment targets for STHs and lymphatic filariasis.

### Role of CMD friends in MDA

For CMDs (44/59) whose friends were involved with MDA, the exact role of those friend groups varied. Each CMD provided at least one role for their friends; 67 responses in total were received from CMDs. The most common to least common role of CMD friends was as follows: spreading information about drug availability (61.02%, 36/59 CMDs), convincing people to swallow drugs (18.64%, 11/59 CMDs), informing CMDs about village problems with MDA (16.95%, 10/59 CMDs), mobilizing the community for MDA (6.78%, 4/59 CMDs), finding people missed for treatment by CMDs (5.08%, 3/59 CMDs), and monitoring CMDs and requiring them to treat everyone in the village (5.08%, 3/59 CMDs).

**Fig. 5:**
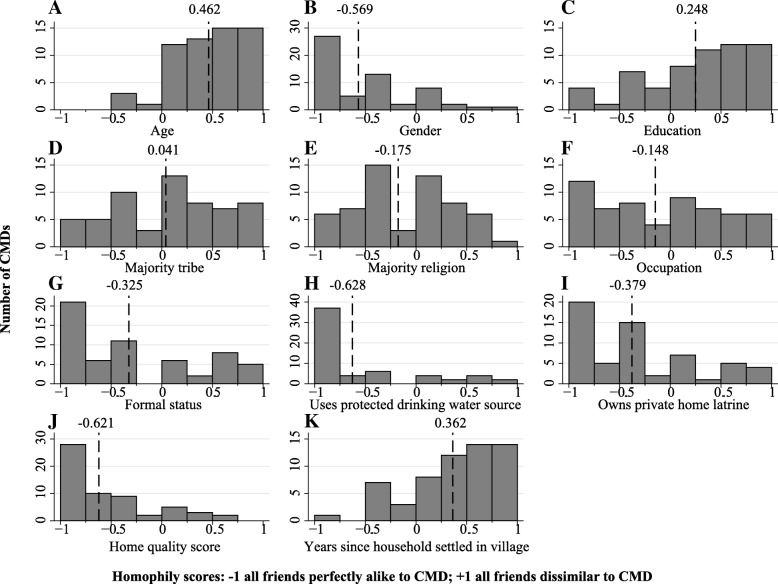
Similarity between community medicine distributors and their friends. Dashed lines show the mean value. All plots include all CMDs (59 obs.). Histogram bar widths = 0.25. **a**–**k** Show CMD homophily scores for all socioeconomic characteristics. Homophily is the measure of likeness for a group of individuals; homophily scores for CMDs reveal to what extent the friend group of a CMD is similar to the CMD of interest when compared by socioeconomic characteristics (Additional file 1: supplementary methods). A score of − 1 indicates perfect similarity, i.e. friends have the same characteristic/value as the CMD for the variable of interest, whilst + 1 indicates perfect dissimilarity, i.e. no friends have the same characteristic/value as the CMD for the variable of interest

To identify who exactly was a friend of a CMD, the composition of CMD friend groups was investigated and compared to the socioeconomic characteristics of the CMD of interest (Fig. 5 and Additional file 1: supplementary methods). On average, CMDs chose to befriend individuals who were of the same gender, had similar formal status in the village, shared preventative health behaviours such as using protected water sources and owning a private home latrine, and were alike with regard to economic wealth as measured by home quality scores. To a much lesser extent, friends also were similar to CMDs concerning religion and occupation. CMDs on average differed from their friends concerning aspects of seniority; friends were dissimilar with respect to age, educational attainment, and the number of years of settlement in the village. Notably, the composition of friend groups did not affect (*p* value > 0.05) CMD treatment rates (Additional file 1: Table S9 and Table S10).

## Discussion

Despite a number of countries with low/stagnant MDA treatment rates [3], WHO resolutions (WHA65.21, WHA50.29, WHA54.19) call for elimination of schistosomiasis, lymphatic filariasis, and STHs as public health problems [25–27]. Current WHO targets for morbidity control are at least 75% treatment of at-risk school-aged children for schistosomiasis and STHs, and for elimination as a public health problem are at least 65% treatment of at-risk school-aged children and adults for lymphatic filariasis. In our study area, treatment rates were well below estimated WHO targets for either controlling morbidity or curbing transmission. For 6779 eligible individuals across 31 villages, the percentage of eligible individuals treated with at least one drug of praziquantel, albendazole, or ivermectin was 44.89%—a notable decrease from 56.77% observed only 3 years prior for MDA in our study villages [17]. Substantial variation in treatment rates amongst CMDs was observed (0–84.25%) and analyzed to profile the best-performing CMDs.

Treatment rates were entirely reliant on CMD characteristics rather than the willingness of the recipient to ingest medicines. CMDs were unpaid volunteers apart from remuneration for training. There is a well-established assumption that volunteers are altruistic [28]. We showed it was incorrect to assume CMDs were altruistic because they were volunteers. Only 13.56% of CMDs were classified as altruistic. These results accord with a previous tracking of MDA, which revealed CMDs are less likely to treat marginalized individuals who are most in need of treatment [15, 19]. Furthermore, we identified inactive CMDs within MDA programmes. The CMDs who did not treat anyone shared a common characteristic—none were classified as altruistic, i.e. having prosocial tendencies. MDA programmes may want to investigate the replacement of inactive CMDs. The CMDs who treated no one or who were in the bottom 50^th^ percentile of treatment rates were serving as CMDs, respectively, for an average of over 5 and 8 years. If these CMDs were inactive during the whole time period that they were serving as CMDs, then these individuals might have undermined disease control. In our study villages, the high prevalence of *S. mansoni* and hookworm has persisted despite over a decade of MDA. There are two strands of research required to identify the conditions of CMD replacement or proactive elimination. First, it remains an open question as to what treatment rate cutoffs should be considered as an indicator of poor performance for individual CMDs or conversely as the treatment targets to be achieved by individual CMDs for disease control. The conservative cutoff of a treatment rate of zero might be too low for MDA programmes to achieve WHO targets. Second, future work should investigate the use of prospective surveys to assess CMD altruism in order to potentially actively eliminate the CMDs without an intrinsic inclination/motivation to help other individuals.

Here, we focused on the observed performance of CMDs in order to provide, to our knowledge, the first measure of treatment rates for individual CMDs and the determinants of those treatment rates. In contrast, other works in our study area have highlighted the challenges faced by CMDs from their perspectives [11, 29], although the correlation with treatment rates was not examined. Addressing the perspective of CMDs was beyond the scope of this study, in particular to avoid interfering with the tracking of routine MDA. However, our findings present new avenues for future research. There might be a need to assess whether or not the characteristics of best/poor-performing CMDs are representative of nuanced challenges that vary between CMDs. In this light, our methodology for measuring treatment rates for individual CMDs may be applied to MDA in other geographic contexts. Afterwards, qualitative surveys may be used to examine how challenges vary from the perspective of CMDs and whether or not such challenges are associated with the profiles of the best-performing CMDs. Previous research in our study area suggests that such profiles may be indicative of the (lack of) social biases of CMDs towards MDA recipients [15]. Apart from the perspective of CMDs, there are challenges that face all CMDs in our study area: (1) no payment despite remuneration for training and hence opportunity costs for being a CMD, (2) the same level of perhaps limited monitoring by district officials, (3) a limited timeframe for MDA that is recommended by the national programmes, and (4) additional possibly conflicting health responsibilities as all CMDs are part of the village health team [11, 17, 29]. Importantly, one directly observed challenge for six CMDs was having a partner CMD who treated no one. Despite this impressive challenge, two out of six of the CMDs were able to still treat at least 60% of eligible individuals within their villages, which is in the 90^th^ percentile of CMD treatment rates. To overcome challenges common to all CMDs, our results showed that the best-performing CMDs involve their close friends to help with MDA-related tasks.

For schistosomiasis, two aspects of MDA implementation are considered formidable problems: treatment of high-risk groups and the distribution of praziquantel. Fishermen/fishmongers, who have high schistosome exposure through frequent freshwater contact, have been suggested as ignored in MDA [30]. Here, we showed that fishermen/fishmongers not only were active CMDs, but also were likely to treat the most people. These findings suggest that selecting CMDs within groups at high-risk of endemic NTDs may increase treatment rates. Amongst all medicines distributed with MDA, praziquantel often is purported as the “showcase” drug for adverse events or poor taste and, in turn, is assumed to result in low rates of administration by CMDs or ingestion by MDA recipients [29]. These common conjectures were not upheld when praziquantel treatment rates were tracked during 1 month of MDA. Praziquantel was most often administered (40.99%) by CMDs when compared to albendazole (36.29%) or ivermectin (29.74%). Only 1.28% (31/2424) of eligible individuals had refused to ingest praziquantel when offered. Moreover, a maximum of 0.37% (9/2424) of eligible individuals had refused to ingest praziquantel due to bad side effects or bad smell/taste. These findings indicate that the causes of low praziquantel treatment rates should not be considered as unique when compared to other drugs administered with MDA. In the context of integrated MDA where multiple NTDs are treated, as was the case in our study, we found no support to indicate that praziquantel should not be administered before other drugs. Consuming praziquantel first did not have a negative impact on subsequent participant compliance with albendazole or ivermectin. Concerning treatment rates, the distribution of praziquantel was positively associated with albendazole and ivermectin treatment rates. Hence, there was no evidence that CMDs experienced any difficulties with praziquantel administration that affected the subsequent distribution of albendazole or ivermectin.

Gender parity is central to MDA programmes [31]; national programmes instruct villages to select one male and one female CMD. Across our study villages, gender balance was observed amongst CMDs (50.85% female). There is a need to scrutinize why gender balance is a requirement of community-based MDA. Being female was associated with 11.96% fewer individuals treated. An inability/unwillingness to treat the opposite sex could not explain the lower treatment rates achieved by female CMDs; 49.76% of the people treated by female CMDs were male. Future research might investigate cultural barriers, such as child-rearing duties, which prevent female CMDs from treating more individuals. This analysis will not be straightforward. Female CMDs may have less time for MDA perhaps due to their domestic duties. However, in our study, this aspect might already have manifested in who ultimately became a CMD, assuming that older women have fewer child-caring responsibilities. The average age of female CMDs was 40.40 years (std. dev. 9.38), which was old relative to other females in the study area (87^th^ percentile of age). Even with the assumption that child-rearing or other responsibilities hinder the performance of female CMDs, social norms exist within our study area where the women are responsible for the medical care of their family members [32]. Such norms might be extended to the village and act as a counter pressure against familial duties, thereby encouraging female CMDs to be high performers in order to meet community expectations. An open question remains concerning whether the balance of CMD characteristics affects MDA and whether having a criterion for CMDs based on gender is justifiable for purposes not related to increasing treatment rates such as empowering women.

In accord with previous MDA tracking in our study area [15, 17, 19], we questioned the proposed capacity constraints of CMDs [9, 11]. It has been suggested that more than two CMDs per village are needed for MDA [9, 11]. Yet, we found that 10.17% (6/59) of CMDs did not treat a single person during 1 month of MDA. There is a possibility that CMDs who treated few or no individuals were engaged with other tasks required for MDA such as sensitizing/mobilizing the community, registering households, or disseminating information about drug availability. However, over 80% of the untreated eligible individuals did not know that drugs were available during our study period. This result suggests that it is highly unlikely that we have omitted MDA-related tasks that would increase/change the CMD’s contribution (and workload) beyond that measured here with treatment rates. Previous work in our study area corroborates this assumption; CMDs were found to register and mobilize individuals and households not before treatment as trained by the national programme, but at the time of treatment [17]. Furthermore, no support was found to explain the uneven CMD contributions as attributable to a negotiation between CMDs about how to split MDA work; we controlled for village-level variation that would explain individual-level CMD variation. Future research is needed to address what combination of CMDs’ attributes results in one CMD not performing MDA duties. Additional studies should examine whether imbalances in social status between CMDs affect which CMD administers treatment. Little is known about how CMD interactions and the balance of CMD personal characteristics affect village-level treatment rates.

Friends of CMDs influenced MDA treatment rates [17]. CMDs who involved their friends with drug distribution were estimated to treat 8.43% more people when compared to CMDs whose friends did not help with MDA. The value placed on CMD friends’ opinions also affected treatment rates. In-group bias was associated with a 13.88% increase in treatment rates when compared to CMDs without in-group biases. The exact structure of CMDs’ friendships did not affect treatment rates. This result differs from findings at the village level [17] due to the limitation of the network approximation used here; only CMD-nominated friendships (outgoing connections) were examined as opposed to full village networks. These partial networks were denser and less varied than actual CMD networks extracted from complete village networks [17]. The influence of the friends of CMDs on treatment outcomes may suggest a need to amend what constitutes a “village affair” for MDA. The selection of CMDs through community-wide meetings is seen as a key driver of empowering local ownership of MDA and ideally increasing treatment rates. Only 44.07% of CMDs were selected through community-wide meetings, whereas 50.85% were selected directly by the local council (village government). The selection of CMDs through community-wide meetings versus other methods of local selection had no association with treatment rates. This result suggests that community-wide meetings are not necessarily a key component of turning MDA into a village affair. In our study, the friends of high-performing CMDs assisted with turning MDA into a village affair. These friends played a key role in informing individuals about available treatment. A better informed target population has greater capacity and better communication channels to provide feedback to CMDs [17]. Importantly, CMDs with friends who were involved with MDA were estimated to treat more individuals. MDA programmes should consider encouraging nominations of CMDs with supportive friendship groups and formally including friends of CMDs during MDA training. Informing the friends of CMDs of ongoing MDA and requesting that the friends help disseminate information and mobilize the community would be an easily testable and inexpensive intervention.

We presented, to our knowledge, the first methodology and initial empirical results to evaluate individual CMDs. Future work may build on our analysis by aiming to track CMD performance over several years. Annual cross-sectional monitoring of CMDs might be conducted through sentinel site surveys in select representative communities. These surveys may enable the observation of any time-varying aspects of CMD performance over consecutive rounds of MDA. There also remains a need to evaluate drug distributor performance in other MDA settings. The 31 villages studied here do not capture the breadth of contexts where MDA for NTDs is ongoing in Sub-Saharan Africa. We studied (1) a rural context with high transmission of an endemic NTD that has received repeated annual MDA, (2) a mature community-based and integrated MDA programme, and (3) door-to-door drug distribution by village-selected CMDs. There remains a need to measure the performance of CMDs, other volunteers, or employed government health workers who conduct MDA in other geographic, programmatic, and NTD settings. Potential aspects of MDA that remain to be studied for drug distributor performance include urban environments, low transmission areas, immature/new MDA programmes, non-integrated MDA, and areas using predominantly central point (drug collection/pick-up) distribution methods. If our findings are reproduced overtime in various settings, then a profile with the shared characteristics of high-performing CMDs might be established for a typical MDA programme for NTDs.

## Conclusions

Evidence-based guidelines for CMD selection and monitoring are needed. Here, we questioned key assumptions of community-based MDA and showed that CMD characteristics and CMDs’ involvement of friend groups may be used to better select and monitor CMD performance. Our findings also revealed two methodological approaches that may simplify MDA evaluation. The percentage of households and individuals treated was nearly perfectly positively correlated, and all results were robust at both household and individual levels. This methodological finding suggests that household-level outcomes measuring treatment of at least one eligible person in the home may be used to approximate individual-level treatment. Treatment rates by individual CMDs should now be evaluated. In our study, all respondents knew which CMD offered drugs to whom, suggesting that the collection of information concerning who treated who may be easily incorporated into routine MDA monitoring. To increase treatment rates with community-based MDA, friends of CMDs should be involved with drug distribution and national programmes should help guide endemic communities to evaluate current CMDs and to replace poor-performing CMDs.

## Additional file

Additional file 1: Supplementary methods include household sampling, MDA tracking and treatment outcomes, intrinsic influence variables, status-seeking variable, CMD selection variable, CMD friends’ role in MDA variable, years as CMD variable, socioeconomic variables, homophily variables, network construction, and network variables [33–54]. **Table S1.** Paired *t* tests comparing individual vs. household treatment outcomes. **Table S2.** Friends not interviewed. **Table S3.** Network variables of community medicine distributors. **Table S4.** Determinants of percentage of the individuals treated without betweenness outlier. **Table S5.** Determinants of percentage of the households treated. **Table S6.** Determinants of percentage of the households treated without betweenness outlier. **Table S7.** Drug-specific models. **Table S8.** Drug-specific models with homophily variables. **Table S9.** Determinants of percentage of individuals treated with homophily variable. **Table S10.** Determinants of percentage of households treated with homophily variable. (PDF 196 kb)

## Abbreviations

CMD: Community medicine distributor
Lasso: Least absolute shrinkage and selection operators
MDA: Mass drug administration
NTDs: Neglected tropical diseases
STHs: Soil-transmitted helminths
WHA: World Health Assembly
WHO: World Health Organization
