# Supplementary material for: Profiling the best-performing community medicine distributors for mass drug administration: a comprehensive, data-driven analysis of treatment for schistosomiasis, lymphatic filariasis, and soil-transmitted helminths in Uganda
Source: BMC Med. 2019 Mar 28;17:69. doi: 10.1186/s12916-019-1303-z (PMC6437990; doi:10.1186/s12916-019-1303-z)
Supplement: Supplementary file 1 — Supplementary methods include household sampling, MDA tracking and treatment outcomes, intrinsic influence variables, status-seeking variable, CMD selection variable, CMD friends’ role in MDA variable, years as CMD variable, socioeconomic variables, homophily variables, network construction, and network variables [33–54]. Table S1. Paired t tests comparing individual vs. household treatment outcomes. Table S2. Friends not interviewed. Table S3. Network variables of community medicine distributors. Table S4. Determinants of percentage of the individuals treated without betweenness outlier. Table S5. Determinants of percentage of the households treated. Table S6. Determinants of percentage of the households treated without betweenness outlier. Table S7. Drug-specific models. Table S8. Drug-specific models with homophily variables. Table S9. Determinants of percentage of individuals treated with homophily variable. Table S10. Determinants of percentage of households treated with homophily variable. (PDF 196 kb) [file 12916_2019_1303_MOESM1_ESM.pdf]

Additional file 1 for "Profiling the best-performing community medicine distributors for mass drug administration: a comprehensive, data-driven analysis of treatment for schistosomiasis, lymphatic filariasis, and soil-transmitted helminths in Uganda"

Goylette F. Chami*, Narcis B. Kabatereine, Edridah M. Tukahebwa

Table of Contents

Supplementary methods 2
Table S1 Paired t-tests comparing individual vs. household treatment outcomes 8
Table S2 Friends not interviewed 9
Table S3 Network variables of community medicine distributors 10
Table S4 Determinants of % of individuals treated without betweenness outlier 11
Table S5 Determinants of % of households treated 12
Table S6 Determinants of % of households treated without betweenness outlier 13
Table S7 Drug-specific models 14
Table S8 Drug-specific models with homophily variables 15
Table S9 Determinants of % of individuals treated with homophily variable 16
Table S10 Determinants of % of households treated with homophily variable 17

SUPPLEMENTARY METHODS

Household sampling

The procedure for sampling households to measure treatment outcomes and to collect household survey data was the following. Exactly 40 households were sampled from each village prior to collecting network data. In each of the 31 villages, we requested the village register, which is a list of all current households in chronological order of when the household moved to the village. Using systematic random sampling, 40 households were selected from the village register. After the random starting point, the interval of household sampling depended on the size of the village. For example, for a village of 120 households, every 3rd household was sampled. If a household was unavailable during the survey period or moved out of the village then the next household in the register (possibly returning to the beginning of the list) was sampled using the interval selection. If village registers were unavailable, national treatment registers were used. These registers provide a list of all households in the village, which was used to record drug distribution during mass drug administration (MDA). Overall, 77.42% (24/31) and 19.35% (6/31) of villages, respectively, utilized village or treatment registers. One village utilized a listing of households for the village voter registration. Once a household was sampled, data was collected on all individuals aged 1+ y/o in that household. In total, for the 31 study villages, household surveys were conducted for 1,240 households with 6,917 people aged 1+ years. The average household size was 5.58 (std. dev. 2.75).

Household data also were collected for friends of community medicine distributor (CMDs), including those friends who were not selected the previous day through systematic random sampling. As a result, in addition to the aforementioned 1,240 households selected with systematic random sampling, we interviewed 173 households with 1,052 people aged 1+ y/o.

For each village, the total number of households was recorded from the village registers (12 villages), the village secretary's records (7 villages), or a research assistant walking around the village to physically count each home (12 villages). The average village size was ~240 homes (std. dev. 121.49; range 87-535). There were 7,452 households across the 31 study villages. Considering this information and the average number of people per home for the random sample of households, ~41,582 people resided in our 31 study villages and thus, were at-risk of helminth infection.

MDA tracking & treatment outcomes

The lead wife and household head were interviewed and asked to provide information about treatment outcomes, i.e. pills offered and ingested, for all members of their household. The definition of a household in our study area is described in Chami *et al.* 2017[17]. We presented drug photos, described the drug purpose and appearance, and physically handed pills/tablets of praziquantel, albendazole, and ivermectin to the study respondents to smell, see, or taste. This approach for eliciting treatment outcomes is described in detail, including its validation for minimizing recall bias from a previous round of MDA in Chami *et al* 2017[17].

The percentage of eligible individuals and the percentage of eligible households that were treated by each CMD were calculated. An individual was classified as treated if the individual was eligible for treatment, was offered at least one drug by a CMD, and had ingested at least one of the drugs offered. This definition accords with the World Health Organization (WHO) definition of surveyed coverage[33]. At the household level, a

household was classified as treated if the household had at least one eligible individual who was offered at least one drug by a CMD and who ingested at least one of the drugs offered. Household treatment status was measured because CMDs were instructed during routine, annual training for MDA to administer treatment by moving from door to door in the village. Moreover, treatment status is positively correlated within a household and predicted by household-level socioeconomic factors[15, 19]. Household treatment indicators also capture key aspects of drug delivery by measuring how often CMDs approach homes[17]. All CMDs were trained on directly observed therapy (DOT)[34], i.e. watching someone swallow pills, yet there was no collection of data by the national programme to verify CMDs conducted DOT. No researchers were present during routine MDA to avoid biasing CMD behaviours, so no data on DOT practices were collected as part of this study.

Figure 1 in the main text summarizes the number of individuals and households included in treatment outcome calculations. Amongst individuals and households randomly sampled, 98.00% (6779/6917) and 99.84% (1238/1240) were eligible for treatment and had no missing data concerning drug offer and ingestion. Within a village, CMD differences in the percentage of eligible individuals treated are unlikely to be influenced by the treated household size. For villages where both CMDs treated at least one person each, there was no significant difference between CMDs concerning the average number of people per treated household (Obs. 24. paired t-test statistic=1.10, avg. 6.03 (std. dev. 1.28) vs. 5.68 (std. dev. 1.05), p-value=0.2816). We observed that if one CMD treated more households than the other CMD then it always was the case that more individuals also were treated by the CMD who treated more households. For an individual CMD, the percentage of households treated was significantly (p-value<0.001) greater than the percentage of individuals treated (Table S1).

Intrinsic influence variables

Prosocial behaviour game

Modified dictator games were used to assess the prosocial/altruistic tendencies of CMDs; the games are shown in the main text Tables 1-2. CMDs were asked to make a choice amongst three options of pre-divided sums of money. Each option had an amount of money that the CMD would receive and the amount of money another individual would receive. Each option represented a different social value orientation[23]. The option with the highest joint payoff (total amount of money) and most equal distribution was the altruistic choice. The option with the highest payoff for the CMD was the egotistic 'selfish' choice. And, the option with the lowest payoff for the other individual (not CMD) was the competitive choice. CMDs were asked to play each game six times. The number of altruistic choices was counted and, as described in van Lange[23], if a CMD chose an altruistic response at least 5/6 times then the CMD was classified as an individual with altruistic tendencies. All games were conducted after the household and network surveys, so both drug distribution and CMD local network structure was not affected by the game. Choice sets were scrambled.

The CMD was in complete control of the choice. We informed CMDs that the other individual did not know who was sending the money, could not find out about the identity of the CMD, and could not respond to the sum of money received, i.e. cannot reject/accept/negotiate or interact in any way with the CMD. Practice games were conducted and a basic mathematics (adding/subtracting) test was given to CMDs to ensure no differences were due to comprehension. The mathematics needed to discern the differences between each choice were incredibly simple and understood well by all CMDs; there were no choices where CMDs needed to 'carry over' for addition or subtraction.

CMDs played modified dictator games where the other individual was a stranger from outside of the village or a close friend. No other information as to the identity of the recipient was given to the CMD other than the category—stranger or close friend. Each game (set of six choices) was shown alone on a page to preclude CMDs from copying choices.

In addition to the information above, the following prompt was provided to CMDs.

> "Now, we would like to understand how you make decisions. In this set of questions, I will ask you to imagine that you have been paired randomly with another person. You must decide among 3 hypothetical choices to distribute UG Shillings. Your choice will decide how many shillings you will get and how many shillings the other person will get. We have divided the UG shillings and you choose the option that is best for you. The more shillings you get, the better for you. The more shillings the other person gets, the better for them. There are no right or wrong answers. Choose the best and most preferred option for you. Although, these are hypothetical questions, I want you to make your choices as if you will actually receive a payment. Now, I am going to ask you to make these choices by imagining that you are paired with different types of people."

Baseline altruism variable

Baseline altruism is a binary variable and equal to one if the CMD was classified as altruistic in the game with a stranger from outside the CMD's village. CMDs were informed that the stranger was a person the CMD will never meet.

In-group bias variable

In-group bias was defined as a preference towards individuals within the CMD's social group (friends) as compared to individuals outside of the CMD's social group (strangers). Quantitatively, in-group bias was measured as a binary variable that is equal to one if the CMD was classified as altruistic to friends, but not classified as altruistic to strangers. Hence, there are three scenarios where in-group bias was equal to zero: if the CMD was 1) altruistic to both friends and strangers, 2) not altruistic to friends but altruistic to strangers, or 3) not altruistic to both friends and strangers.

Status-seeking variable

The status-seeking behaviour of CMDs was investigated because CMDs choose to volunteer to distribute drugs during MDA to gain status in their village[15]. For each friend that was nominated by the CMD, we asked whether the CMD purposely sought that edge (connection). The formal status of the friends, capturing powerful positions within each village, was measured as described below in the socioeconomic variables section. If a friendship was purposely sought and was to a person with formal (high) status in the CMD's village then that edge was classified as a status-sought edge. Image association is equal to one if the CMD had at least one status-sought edge.

CMD selection variable

Although national MDA programmes instruct communities to select CMDs through a village-wide meeting and open nomination, CMDs may be directly appointed by other means[35]. CMD selection is a nominal variable with three categories: community meeting, appointment by someone from the local council (village government), and internal selection by members of the village health team (bottom tier of Ugandan health care system).

CMD friends' role in MDA variable
CMDs were asked if their friends had any role in drug distribution during MDA. The friends' role in MDA was coded as a binary variable that is equal to one if the CMD stated they receive help from friends during MDA. Such help/roles included spreading information about drug availability, telling CMDs about MDA problems, finding people missed for treatment, convincing people to swallow drugs, monitoring and requiring CMDs to treat everyone, and mobilizing the community.

Years as CMD variable
'Years as CMD' is a count of the number of years the CMD had been distributing drugs through MDA. If a CMD indicated more than 14 years then the response was recoded as 14. At the time of our study in 2016, MDA had been ongoing in Mayuge District since 2003.

Socioeconomic variables
Eleven socioeconomic variables were constructed for all (59) CMDs and their 278 friends. There are seven individual-level variables. Age is a continuous variable and measured in years. Gender is a binary variable that is equal to one if the individual was female. Education is an ordinal variable measured as the highest level of education attained where 0 = none, 1-7 = Primary 1 to Primary 7 respectively, 8-13 = Secondary 1 to Secondary 6 respectively, 14 = Diploma, 15 = some university, and 16 = completed university. Majority tribe and majority religion are binary variables that equal one if the individual belonged respectively to the tribe or religion with the highest percentage of people, as measured by the random sample, from their village. Musoga was the majority tribe for every village except village ID 1 where the majority tribe was Mudama. Also, one village (ID 21) had two majority tribes, i.e. both had the same equally highest share of the village population (here Musoga and Mudama, 31%). Christianity—a distinct classification from born-again Christianity—was the majority religion in all but five villages (IDs 8, 10, 22, 27, and 31) that had a Muslim majority. One village (ID 21) had both Christian and Muslim majority religions (46%). Occupation measured the two main occupations in the study area[17]. It was coded as a nominal variable with three categories: farmer, fisherman/fishmonger, and other job. Individuals only provided one occupation and were asked to provide their main income-earning job. Formal status is a binary variable and equal to one if the individual currently or previously held at least one of 13 village positions including chairman, vice chairman, secretary, defense secretary, gender secretary, disabled secretary, youth councilor, information secretary, elderly secretary, tribe leader, clan leader, religious leader, or beach management team member.

Among the 11 socioeconomic variables, four were measured at the household level. WHO/UNICEF guidelines[36] for classifying access to safe water and sanitation were used to construct binary indicators for household potable water use and ownership of a home latrine. 'Safe water' was equal to one if an individual belonged to a home that used protected sources as its main source of drinking water. In our study villages, protected sources included piped water, village taps, boreholes, or protected wells. Ownership of a home latrine was equal to one if a household had a covered pit latrine with privacy. Home quality score was defined as the sum of scores for materials used to construct the roof, wall, and floor. Materials were ranked from 1-4 and in order were grass/thatch, sticks, plastic, and metal for the roof; mud/sticks, plastic, metal, or bricks/cement for the walls; and mud, plastic, wood planks, bricks/cement for the floor. 'Years in the village' was a count of the number of years since the first person from the household settled in the village.

Homophily variables

We examined how similar CMDs were to their friends based on the previously described 11 socioeconomic characteristics. Homophily was measured as described in Krackhardt and Stern.[37] An external edge was a friend with a characteristic different to that of the CMD and an internal edge was a friend with a characteristic similar/exact to that of the CMD. Homophily was calculated as (E-I)/(E+I) where E = total external edges and I = total internal edges. This indicator varies from -1 to +1 where -1 indicates perfect homophily, i.e. the CMD was only connected to people with the same value of the socioeconomic characteristic, and +1 indicates that the CMD was only connected to people unlike the CMD for the measured characteristic. Gender, majority tribe, majority religion, occupation, safe water, home latrine, and formal status were directly matched to determine if the edge was external. A direct match compared the exact CMD's value to the exact friend's value and any differences were considered an external edge. Education was recoded to have only four categories of none, primary, secondary, and diploma (no CMDs or their friends went to university). This variable was then directly matched for CMDs and their friends to determine external edges. For home quality score, an internal edge was considered to be anything in the range of +3/-3 of the CMD's value. For age and years in the village, an internal edge was measured as anything in the range of +5/-5 of the CMD's value.

Network construction

In each of the 31 study villages, there were two CMDs. We examined the local networks of 59/62 CMDs. Three CMDs were excluded (Table S2). One CMD (ID e02, village ID 20) was not interviewed. For two CMDs (IDs e02, village IDs 24 & 30), none of the close friends nominated were interviewed and consequently the networks could not be constructed. Close friendship (a network edge) was defined as germane to our study area[17].

Close friendship prompt for CMDs:

> Please tell me the clan name first then the second name of up to 10 people that are very close friends to you. You should feel comfortable to turn to this person to borrow tools for fishing or farming without paying. A close friend is also someone that you see frequently. You should trust this person for advice. Do not name anyone in your household. Provide the names in the order of who is your closest friend first. Only name people in your village.

Network nodes were CMDs, or the individuals who were nominated by a CMD and also directly interviewed. When CMDs were asked to name their friends, interviewers did not tell CMDs that the friends would be interviewed the next day. This procedure ensured that CMDs did not provide the interviewer with false contacts. Despite limiting nominations to a maximum of 10 close friends, no upward bias was observed. No CMD nominated 10 individuals. The average number of close friends nominated by all interviewed CMDs was 4.88 (Obs. 61, Range 2-9, std. dev. 1.53). Close friends who were not interviewed were excluded from all node sets (Table S2). Thus, all networks for the same CMD were of the same order, i.e. the same size with corresponding node sets.

Close friends of CMDs were interviewed. Each interviewee was shown a list of people (names) that included both CMDs and all close friends of both CMDs. The list also showed the gender of each close friend and the name and gender of their household head. Interviewees were asked to indicate if they were close friends with each person on the list.

Close friendship prompt for friends of CMDs:

> I am going to show you a list of people. Please tell me which of these people are very close friends with you. A close friend is someone that you should feel comfortable to turn to for borrowing tools for fishing or farming without paying. A close friend is also someone that you see frequently and trust for advice.

Network variables

Networks were analyzed in Python v2.7 with the NetworkX library[38], Stata v13.1, and R v3.2.3 with iGraph v1.0.1[39] and sna v1.13[40]packages. All edges were undirected. Table S3 summarizes CMD network indicators.

Network size

CMD degree was simply equal to the number of nodes each network. Degree was measured since individuals who belong to large networks have been shown to be less prosocial[28]. By reducing network clustering, network size has been found to be negatively correlated with prosocial behaviour[41].

Cohesion

We examined three indicators of cohesion – density, transitivity, and k-components. Density is the fraction of all possible edges that exist in the network; it was calculated after removing the CMD and all CMD edges[42, 43]. Hence, density here was equal to calculating the clustering coefficient for the CMD, which was the fraction of all possible triangles involving the CMD and his/her neighbours[44]. Transitivity was calculated as the fraction of all possible triangles in the network after removing the CMD and all CMD edges[42, 45]. K-components represent cohesive blocks within a network[46]. Here k-components represented the minimum number of nodes that need to be removed to disconnect a network; these k-component numbers represented the hierarchical nested structure of a network. The highest k-component number of a node was therefore a measurement of node embeddedness. The highest k-component number of each node was calculated and the average of the highest k-component number of all nodes was examined for each CMD[47].

Lack of cohesion

Three indicators of sparse networks were measured – effective degree, betweenness centrality, and the number of components. The effective degree (effective network size) was a measure of the lack of structural cohesion/redundancy[48]. The average degree of CMD alters, which excluded edges to the CMD, was subtracted from CMD degree. High betweenness centrality is a requirement for structural holes[48] and was measured (not normalized) as described in Everett and Borgatti 2005[49]. This indicator also was of interest as it has been shown to positively correlate with prosocial behaviour[50-53]. The number of components was a count of connected components and isolates in the network after removing the CMD and all CMD edges. To ensure the number of components was not simply a redundant measure of network size, the number of components was divided by CMD degree. This variable was an indicator of structural diversity, measuring the association of CMDs to distinct social groups[54].

Table S1 Paired t-tests comparing individual vs. household treatment outcomes

Variable	Obs.	Mean	Std. Dev.	95% CI		p-value	t-statistic
Village level							
% eligible individuals treated	31	0.447	0.266	0.350	0.545	<0.001	-7.278
% eligible households treated	31	0.535	0.260	0.439	0.630		
CMD 1							
% eligible individuals treated by CMD e01	31	0.256	0.202	0.182	0.330	<0.001	-4.029
% eligible households treated by CMD e01	31	0.302	0.213	0.224	0.380		
CMD 2							
% eligible individuals treated by CMD e02	31	0.245	0.235	0.159	0.331	<0.001	-5.207
% eligible households treated by CMD e02	31	0.295	0.253	0.202	0.388		

Table S2 Friends not interviewed

Village ID	CMD ID	No. friends not interviewed/ No. not nominated by CMD	Notes
3	e01	1/9	
3	e02	1/3	
4	e01	3/7	
5	e01	1/6	
7	e02	1/5	
13	e01	1/3	
17	e02	1/4	
19	e01	1/10	Same person named as e02
19	e02	1/6	Same person named as e01
20	e01	2/6	
20	e02	unknown	CMD not interviewed
21	e02	1/5	
24	e01	1/6	Same as one person missing for e02
24	e02	4/4	CMD's friends not interviewed
25	e02	1/7	
26	e01	1/7	
27	e02	2/8	
28	e01	1/4	
30	e02	4/4	CMD's friends not interviewed
31	e01	1/6	

Table S3 Network variables of community medicine distributors

Variable	Obs.	Mean	Std. Dev.	Min	Max	50th %ile
No. of close friends (degree)	59	4.712	1.521	2	9	4
No. friendships within CMD's friend group (edges)	59	13.085	7.949	3	42	10
Density	59	0.836	0.221	0	1	0.905
Transitivity	59	0.760	0.334	0	1	0.907
Avg. k-component	59	3.847	1.395	1	7	3.857
Effective degree	59	1.581	0.748	1	5.250	1.500
Betweenness	59	0.732	1.652	0	11.583	0.333
Components	59	0.313	0.342	0.111	2	0.250

CMD = community medicine distributor.

Table S4 Determinants of % of individuals treated without betweenness outlier

Variable[a]	Coef.	Clustered robust SE	p-value	95% Confidence interval	
In-group bias[b]	0.139	0.059	0.025	0.018	0.259
Selected by local council[c]	-0.085	0.054	0.122	-0.195	0.024
Friends help with MDA[d]	0.082	0.039	0.045	0.002	0.162
Female	-0.121	0.056	0.041	-0.236	-0.005
Fisherman/fishmonger[e]	0.145	0.039	0.001	0.064	0.225
Household uses protected drinking water source	0.135	0.040	0.002	0.054	0.216
Network betweenness	0.017	0.028	0.545	-0.040	0.075
Constant	0.103	0.049	0.046	0.002	0.204

Obs. 58

$R^2 = 0.320$

F-stat. = 12.76, *F-stat.* p-value<0.0001

Root mean squared error (RMSE) from 8-fold cross validation = 0.187

Variables selected through Lasso with 8-fold cross validation.

Mean squared error (MSE) of Lasso cross validation = 0.076

[a]The results are from an ordinary least squares regression with standard errors clustered by village.
[b]In-group bias is positive if the community medicine distributor (CMD) was altruistic towards their friends and not altruistic towards strangers.
[c]The base category includes CMD selection by community meeting or direct nomination from a village health team member.
[d]MDA= mass drug administration.
[e]The base category for these occupations includes all other CMD occupations.

One CMD who had network betweenness = 11.58 was removed; the remaining 58 CMDs had network betweenness ≤ 3.

Table S5 Determinants of % of households treated

Variable[a]	Coef.	Clustered robust SE	p-value	95% Confidence interval	
In-group bias[b]	0.161	0.060	0.012	0.039	0.283
Selected by local council[c]	-0.075	0.054	0.180	-0.186	0.036
Friends help with MDA[d]	0.101	0.042	0.023	0.015	0.186
Female	-0.129	0.058	0.034	-0.248	-0.011
Fisherman/fishmonger[e]	0.195	0.053	0.001	0.087	0.302
Household uses protected drinking water source	0.124	0.046	0.011	0.031	0.218
Network betweenness	0.035	0.008	<0.001	0.019	0.051
Constant	0.121	0.055	0.034	0.010	0.233

Obs. 59

$R^2 = 0.355$

F-stat. = 8.63, *F-stat.* p-value<0.0001

Root mean squared error (RMSE) from 8-fold cross validation = 0.193

Variables selected through Lasso with 8-fold cross validation.

Mean squared error (MSE) of Lasso cross validation = 0.078

[a]The results are from an ordinary least squares regression with standard errors clustered by village.
[b]In-group bias is positive if the community medicine distributor (CMD) was altruistic towards their friends and not altruistic towards strangers.
[c]The base category includes CMD selection by community meeting or direct nomination from a village health team member.
[d]MDA= mass drug administration.
[e]The base category for these occupations includes all other CMD occupations.

Table S6 Determinants of % of households treated without betweenness outlier

Variable[a]	Coef.	Clustered robust SE	p-value	95% Confidence interval	
In-group bias[b]	0.161	0.060	0.012	0.038	0.283
Selected by local council[c]	-0.075	0.055	0.180	-0.186	0.037
Friends help with MDA[d]	0.100	0.042	0.023	0.015	0.186
Female	-0.129	0.058	0.034	-0.248	-0.011
Fisherman/fishmonger[e]	0.194	0.052	0.001	0.088	0.300
Household uses protected drinking water source	0.124	0.046	0.011	0.031	0.218
Network betweenness	0.033	0.030	0.277	-0.028	0.094
Constant	0.122	0.056	0.036	0.009	0.236

Obs. 58
$R^2 = 0.342$
F-stat. = 6.46, *F-stat.* p-value=0.0001
Root mean squared error (RMSE) from 8-fold cross validation = 0.195
Variables selected through Lasso with 8-fold cross validation.
Mean squared error (MSE) of Lasso cross validation = 0.078

[a]The results are from an ordinary least squares regression with standard errors clustered by village.
[b]In-group bias is positive if the community medicine distributor (CMD) was altruistic towards their friends and not altruistic towards strangers.
[c]The base category includes CMD selection by community meeting or direct nomination from a village health team member.
[d]MDA= mass drug administration.
[e]The base category for these occupations includes all other CMD occupations.

One CMD who had network betweenness equal to 11.58 was removed; the remaining 58 CMDs had network betweenness ≤ 3.

Table S7 Drug-specific models

Model	Variable	Coef./ Value	Clustered robust SE	p-value	95% Confidence interval	
Outcome: Praziquantel	In-group bias	0.106	0.055	0.051	0.000	0.213
	Constant	0.188	0.035	<0.001	0.121	0.256
	R^2	0.070				
	RMSE	0.194				
	MSE	0.077				
Outcome: Albendazole	In-group bias	0.104	0.051	0.041	0.004	0.205
	Household uses protected drinking water source	0.128	0.042	0.002	0.046	0.210
	Constant	0.053	0.041	0.202	-0.028	0.134
	R^2	0.147				
	RMSE	0.188				
	MSE	0.077				
Outcome: Ivermectin	In-group bias	0.106	0.050	0.032	0.009	0.203
	Selected by local council	-0.102	0.043	0.020	-0.187	-0.016
	Household uses protected drinking water source	0.119	0.042	0.005	0.036	0.202
	Constant	0.076	0.038	0.045	0.002	0.150
	R^2	0.205				
	RMSE	0.169				
	MSE	0.079				

Obs. 59

RMSE = root mean squared error from 8-fold cross validation of model.

Variables selected through Lasso with 8-fold cross validation.

MSE = mean squared error of Lasso 8-fold cross validation.

Breusch-Pagan test of independent equations. Chi^2 = 132.868, p-value = <0.0001.
Standard errors were corrected for the correlation between the error terms of the different models. Models were run as simultaneous equations.

Table S8 Drug-specific models with homophily variables

Model	Variable	Coef./ Value	Clustered robust SE	p-value	95% Confidence interval	
Outcome: Praziquantel	In-group bias	0.111	0.054	0.039	0.005	0.217
	Gender homophily	-0.091	0.039	0.021	-0.168	-0.014
	Constant	0.134	0.045	0.003	0.045	0.223
	R^2	0.121				
	RMSE	0.189				
	MSE	<0.001				
Outcome: Albendazole	In-group bias	0.106	0.051	0.039	0.005	0.206
	Household uses protected drinking water source	0.128	0.041	0.002	0.048	0.208
	Majority tribe homophily	0.042	0.042	0.323	-0.041	0.124
	Constant	0.050	0.041	0.217	-0.030	0.131
	R^2	0.161				
	RMSE	0.190				
	MSE	<0.001				
Outcome: Ivermectin	In-group bias	0.097	0.046	0.035	0.007	0.188
	Selected by local council	-0.100	0.044	0.022	-0.185	-0.014
	Household uses protected drinking water source	0.116	0.043	0.007	0.032	0.201
	Majority religion homophily	0.053	0.045	0.243	-0.036	0.141
	Constant	0.091	0.038	0.018	0.015	0.166
	R^2	0.223				
	RMSE	0.170				
	MSE	<0.001				

Obs. 59

RMSE = root mean squared error from 8-fold cross validation of model.

Variables selected through Lasso with 8-fold cross validation.

MSE = mean squared error of Lasso 8-fold cross validation.

Breusch-Pagan test of independent equations. Chi^2 = 124.363, p-value = <0.0001.
Standard errors were corrected for the correlation between the error terms of different models.
Models were run as simultaneous equations.

Table S9 Determinants of % of individuals treated with homophily variable

Variable[a]	Coef.	Clustered robust SE	p-value	95% Confidence interval	
In-group bias[b]	0.139	0.060	0.028	0.016	0.262
Selected by local council[c]	-0.071	0.052	0.183	-0.178	0.036
Friends help with MDA[d]	0.080	0.037	0.038	0.005	0.156
Female	-0.103	0.061	0.102	-0.227	0.022
Fisherman/fishmonger[e]	0.157	0.045	0.002	0.064	0.250
Household uses protected drinking water source	0.136	0.042	0.003	0.051	0.221
Network betweenness	0.030	0.008	0.001	0.013	0.046
Gender homophily	-0.059	0.054	0.285	-0.169	0.052
Constant	0.046	0.084	0.584	-0.125	0.218

Obs. 59

R^2 = 0.346

F-stat. = 12.66, *F-stat.* p-value<0.001

Root mean squared error (RMSE) from 8-fold cross validation = 0.179

Homophily variables selected through Lasso with 8-fold cross validation.

Mean squared error (MSE) of Lasso cross validation = <0.001

[a]The results are from an ordinary least squares regression with standard errors clustered by village.

[b]In-group bias is positive if the community medicine distributor (CMD) was altruistic towards their friends and not altruistic towards strangers.

[c]The base category includes CMD selection by community meeting or direct nomination from a village health team member.

[d]MDA= mass drug administration.

[e]The base category for these occupations includes all other CMD occupations.

Table S10 Determinants of % of households treated with homophily variable

Variable[a]	Coef.	Clustered robust SE	p-value	95% Confidence interval	
In-group bias[b]	0.163	0.060	0.010	0.042	0.285
Selected by local council[c]	-0.090	0.050	0.083	-0.192	0.012
Friends help with MDA[d]	0.094	0.044	0.040	0.005	0.183
Female	-0.118	0.060	0.060	-0.241	0.005
Fisherman/fishmonger[e]	0.216	0.055	0.001	0.103	0.330
Household uses protected drinking water source	0.128	0.047	0.010	0.032	0.223
Network betweenness	0.033	0.008	0.000	0.017	0.050
Majority tribe homophily	0.048	0.040	0.235	-0.033	0.130
Constant	0.122	0.053	0.027	0.014	0.229

Obs. 59
$R^2 = 0.367$
F-stat. = 8.77, *F-stat.* p-value<0.001
Root mean squared error (RMSE) from 8-fold cross validation = 0.193
Homophily variables selected through Lasso with 8-fold cross validation.
Mean squared error (MSE) of Lasso cross validation = <0.001

[a]The results are from an ordinary least squares regression with standard errors clustered by village.
[b]In-group bias is positive if the community medicine distributor (CMD) was altruistic towards their friends and not altruistic towards strangers.
[c]The base category includes CMD selection by community meeting or direct nomination from a village health team member.
[d]MDA= mass drug administration.
[e]The base category for these occupations includes all other CMD occupations.
